# Integration of constraint-based modeling with fecal metabolomics reveals large deleterious effects of *Fusobacterium* spp. on community butyrate production

**DOI:** 10.1080/19490976.2021.1915673

**Published:** 2021-05-31

**Authors:** Johannes Hertel, Almut Heinken, Filippo Martinelli, Ines Thiele

**Affiliations:** a School of Medicine, National University of Galway, Galway, Ireland; b Department of Psychiatry and Psychotherapy, University Medicine, Greifswald, Germany; c Ryan Institute, National University of Galway, Galway, Ireland; d Discipline of Microbiology, National University of Galway, Galway, Ireland; e APC Microbiome Ireland, University College Cork, Cork, Ireland

**Keywords:** Metabolic modeling, microbiome, fusiobacteria, metagenomic data, flux balance analysis

## Abstract

Characterizing the metabolic functions of the gut microbiome in health and disease is pivotal for translating alterations in microbial composition into clinical insights. Two major analysis paradigms have been used to explore the metabolic functions of the microbiome but not systematically integrated with each other: statistical screening approaches, such as metabolome-microbiome association studies, and computational approaches, such as constraint-based metabolic modeling. To combine the strengths of the two analysis paradigms, we herein introduce a set of theoretical concepts allowing for the population statistical treatment of constraint-based microbial community models. To demonstrate the utility of the theoretical framework, we applied it to a public metagenomic dataset consisting of 365 colorectal cancer (CRC) cases and 251 healthy controls, shining a light on the metabolic role of *Fusobacterium* spp. in CRC. We found that (1) glutarate production capability was significantly enriched in CRC microbiomes and mechanistically linked to lysine fermentation in *Fusobacterium* spp., (2) acetate and butyrate production potentials were lowered in CRC, and (3) *Fusobacterium* spp. presence had large negative ecological effects on community butyrate production in CRC cases and healthy controls. Validating the model predictions against fecal metabolomics, the *in silico* frameworks correctly predicted *in vivo* species metabolite correlations with high accuracy. In conclusion, highlighting the value of combining statistical association studies with *in silico* modeling, this study provides insights into the metabolic role of *Fusobacterium* spp. in the gut, while providing a proof of concept for the validity of constraint-based microbial community modeling.

## Introduction

The gut microbiome, with its trillions of bacteria, contributes crucially to human metabolism in health and disease.^1^ It generates otherwise inaccessible nutrients,^2^ inactivates and activates drugs,^3^ and produces potentially harmful metabolites.^4^ Recent advances in sequencing techniques have given rise to a wealth of insights into gut microbiome composition patterns, revealing that the gut microbiome is affected in many human diseases.^5^ Besides results stemming from observational human cohort studies, an impressive number of experimental studies on animal models have resulted in insight into the mechanisms by which the gut microbiome interacts with the host organism.^6^

Specifically, bacterial fermentation pathways play a key role in mediating host-microbe metabolic interactions. Short-chain fatty acids (SFCAs), namely acetate, butyrate, and propionate, arise from gut microbial fermentation of dietary fiber.^7^ Microbial fermentation of protein also results in SFCA production but mostly results in branched-chain fatty acids, such as isobutyrate, 2-methylbutyrate, and isovalerate.^8^ SFCAs, especially butyrate, directly modulate host physiology by serving as signaling molecules.^7^ For instance, they act as histone deacetylase (HDAC) inhibitors, inducing the expression of the tumor suppressor gene *CDKN1A*.^9^ Additionally, SFCAs bind to G protein-coupled receptors (GPCRs),^10,11^ playing a role in the pathogenesis of colorectal cancer (CRC).^12,13^ Butyrate is protective against CRC since it is both a potent antitumor and anti-inflammatory agent^14^ that is mediated by its HDAC-inhibiting effects.^15^ Moreover, butyrate serves as the main carbon source for healthy colonocytes but not for tumor cells.^7^ Multiple studies have consistently reported a decrease in butyrate-producing bacteria in CRC patients.^7^ However, the general metabolic consequences of compositional changes in CRC-related microbiomes remain unclear.

The fecal metabolome is considered to be a readout of the functional capabilities of the gut microbiome.^16,17^ Consequently, changes in fecal metabolome profiles in CRC have also been linked to altered microbial abundance patterns via statistical association studies.^18–20^ However, it remains challenging to identify the mechanisms by which the microbiome changes the metabolome, as statistical associations may be caused by indirect effects and confounding.^21,22^ Therefore, correlations between fecal metabolites and species abundances persist as cryptic in terms of their biological meaning. Moreover, as species share metabolic capabilities and functions even across different phyla,^23^ it is by no means clear that a change in composition will result in a change in metabolic function. Hence, two gut microbial communities may look drastically different regarding their species composition, while they may be largely equivalent in terms of metabolic functions, complicating interpretations of metagenomics studies. As the gut microbiome acts as a complex ecosystem where species have to be understood in their role within communities, systems biology approaches seem to be best suited to tackle the problem of translating patterns of species abundance into patterns of metabolic function.^21^ Similarly, unraveling the complex ecological interdependencies within microbial communities in terms of metabolic functions seems to be hardly achievable by statistical approaches such as correlation analyses, pathway enrichment analyses, or machine learning alone.^24^

In addition to statistical approaches to explore the metabolic functions of microbial communities, various computational approach 4es from small-scale ordinary differential equations (ODEs)^25^ to the large-scale efforts of genome-scale reconstructions^23^ have been developed. In particular, the framework of constraint-based modeling and reconstruction analysis (COBRA) has proven to be useful for the interrogations of metabolic functions of microbial communities.^26,27^ COBRA represents a scalable systems biology computational modeling approach that is widely applied in the field of microbiome research.^28–30^ Its strengths of integrating genomic data with condition-specific constraints are specifically designed to deliver on the task of characterizing the metabolic functions of microbial communities.^31^ Importantly, in contrast to pure statistical approaches, COBRA allows for the mechanistic examination of microbe-metabolite relations within one community.^32^

However, with the tools for personalizing COBRA community models via microbial abundance data,^33^ the possibility has emerged to explore populations of COBRA community models across strata of individuals, such as those represented in case-control studies.^34,35^ Until now, only a few studies have explored the field of such population-based COBRA community modeling, employing statistical screening approaches for *in silico* biomarkers in the case of Parkinson’s disease.^34,35^ However, comprehensive theoretical frameworks that make use of the full information encoded in COBRA models for research at the population-level have not been developed so far.

In response to these challenges, we present a set of theoretical concepts integrating statistical approaches to microbiome research with COBRA community modeling, thereby broadening the methodological arsenal of the microbiome research community. Additionally, the application of the introduced conceptual frameworks results in predictions, which can be directly tested via statistical integration of metagenome and fecal metabolome data.

Applying our frameworks to a recently published metagenomics dataset of a colon cancer case-control study,^16^ we successfully validated our frameworks by integrating them with fecal metabolomic measurements from the same study. In this way, we utilized the theoretical concepts to identify the unique metabolic capabilities of *Fusobacterium* spp. in conjunction with unraveling their deleterious effects on community butyrate production. Crucially, we demonstrate that our theoretical frameworks rooted in COBRA community modeling can correctly predict the *in vivo* species-metabolite association patterns for butyrate and glutarate. Therefore, we demonstrate the validity of COBRA community modeling in a proof-of-principle analysis, providing novel insights into the role of *Fusobacterium* spp. in CRC.

## Results

**Figure 1.**
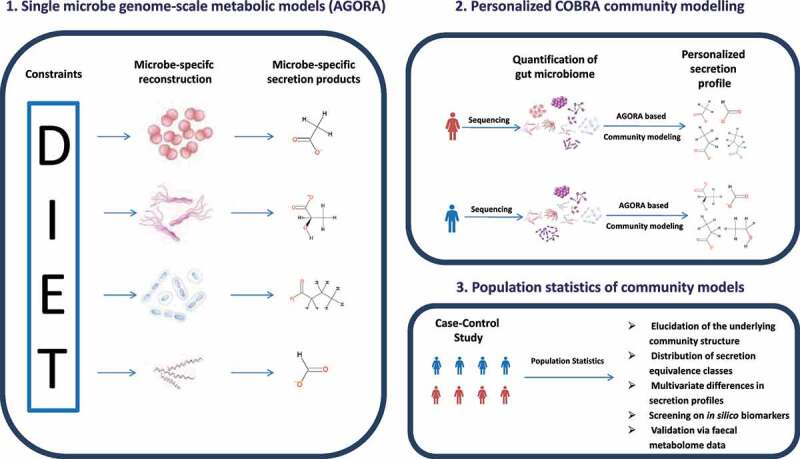
Overview over the three levels of AGORA-based community modeling used in this study. The concepts displayed in Table 2 refer to the population level

To translate microbiome abundance patterns into patterns of metabolic functions, we applied community modeling to a colorectal cancer (CRC) case-control cohort,^16^ which included 616 individuals (365 CRC cases and 251 healthy controls), with metagenomic data (Table 1). For each individual, a personalized microbiome model was built, consisting of 80,343.84 (SD = 19,936.36) reactions on average, appropriately contextualized with a simulated average Japanese diet, and subsequently interrogated through flux balance analysis simulations (Methods). The simulations resulted in one model producing nothing, indicating an infeasible model specification. This case was excluded from the analyses. The resulting personalized flux profiles were then analyzed in the context of clinical parameters and metabolomic findings through population statistics modeling. Thus, this study utilized three distinct levels of modeling (Figure 1a): (1) The strain-specific AGORA genome-scale metabolic reconstructions, (2) the personalized COBRA community models integrating diet data and the individual’s metagenomic data resulting in individual flux profiles, and (3) the statistical modeling of populations of community models. Note that the first two steps are deterministic, while the third step is statistical (Table 2).

**Table 1.** Sample characteristics of the study

	CRC patients (*n* = 364)	Healthy controls (*n* = 251)	*p*-Value
Age, mean (SD)	62.4 (9.91)	60.81 (12.64)	0.095^a^
BMI, mean (SD)	22.95 (3.57)	22.67 (3.04)	0.294^a^
Female, %	39.29%	45.82%	0.115^b^
Stage of the disease	HS, 10.99% MP, 18.41% Stage 0, 19.78% Stage I/II, 30.49% Stage III/IV, 20.33%	NA	NA
Species richness, mean (SD)	69.74 (18.33)	63.91 (15.96)	<0.001^a^
# Metabolites produced, mean (SD)	157.06 (6.67)	156.17 (7.20)	0.123^a^
# Reactions in community models	83010.27 (20707.52)	76477.00(18115.58)	<0.001^a^
# Unique reactions in community models, mean (SD)	2896.80 (99.58)	2885.28 (105.29)	0.190^a^

CRC = Colorectal cancer, SD = Standard deviation, HS = Healthy after surgery, MP = Multiple polyps, ^a^*p*-value from Welch t-tests, ^b^*p*-value from Fisher’s exact test.

**Table 2.** Theoretical concepts used in this study

Theoretical concept	Description^a^	Type of modeling
Net metabolite production capability	The possibility to produce a metabolite	Deterministic
Net metabolite production capacity	The amount of a metabolite (mmol/d), which can be maximally produced	Deterministic
Direct species production effect	The average contribution of a species to the net metabolite production capacity of a community	Statistical
Ecological species effect	The difference between average net metabolite production capacities of communities with a species and communities without a species after discounting the direct species net production capacity	Statistical
Metabolic Equivalence	Equivalence of two communities in terms of net metabolite production capabilities	Deterministic
Metabolically Sufficient	A species/reaction is called sufficient for a metabolite, if presence of the species/reaction within a community means that the metabolite can be produced.	Deterministic
Strictly metabolically sufficient	A species/reaction is called strictly sufficient if it is sufficient given all other sufficient species/reactions.	Deterministic
Metabolically necessary	A species/reaction is called necessary for a metabolite, if absence of the species/reaction within a community means that the metabolite cannot be produced	Deterministic
Strictly metabolically necessary	A species/reaction is called strictly necessary, if it is necessary given all other necessary species/reactions.	Deterministic

^a^Formal definitions can be found in the Methods section. All definitions are conditional on the applied diet constraints.

### Microbial communities are unique in their metabolic capabilities in healthy controls and CRC patients

To gain insight into the distribution of gut microbial metabolic capabilities across samples, we explored the distribution of secretion patterns in CRC cases and controls using the concept of metabolic equivalence (see Methods). We call two communities equivalent regarding a certain set of metabolites, if the subset of metabolites with a net production capacity greater than zero conditional on a common diet is the same for both communities. In the AGORA resource,^23^ the net production capacity calculation for all 413 metabolites that are associated with exchange reactions^36^ was possible, resulting in the theoretical number of 2^413^ different equivalence classes for the whole set of metabolites. However, from these 413 metabolites, 224 metabolites were produced by no model under the given constraints and 90 metabolites by all models, meaning that the secretion capability of 99 metabolites showed variance across the microbiome community models, with 43 metabolites being produced by at least 5% of the models and maximally 95% of the models (Table S1). Despite this high level of overlapping of metabolic capabilities between microbiome models, we detected 607 different equivalence classes in 615 simulated communities. Hence, microbial communities are mostly metabolically unique in their profiles of metabolic capabilities, thereby contributing to the individuality of human metabolism in health and disease.

### *Glutarate production capability is enriched in CRC cases and unique to* Fusobacterium *sp.*

**Figure 2.**
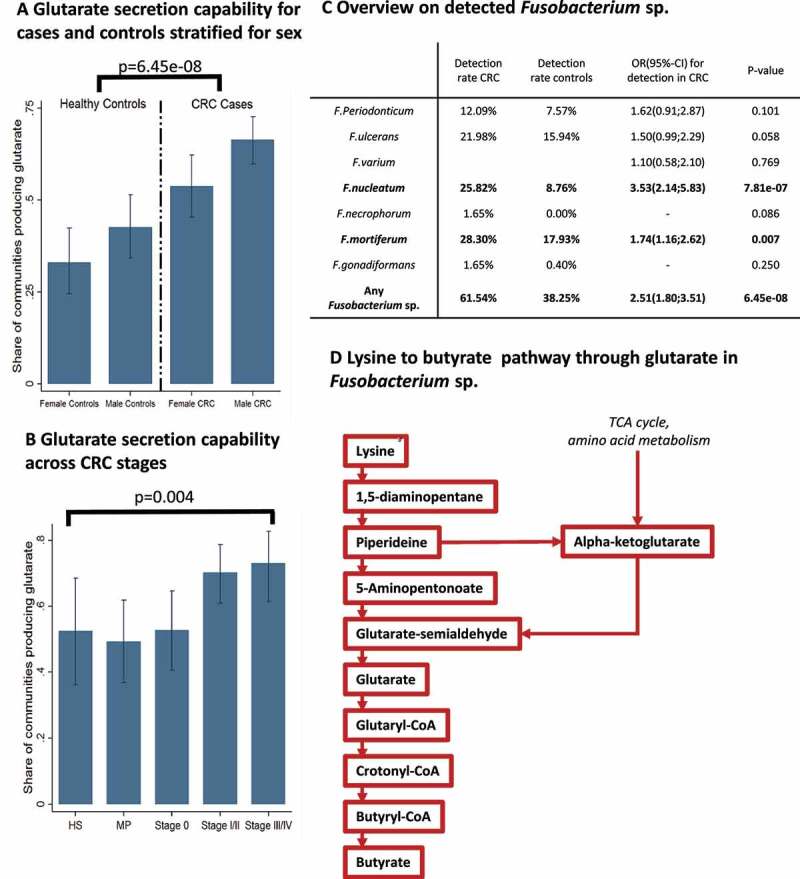
Glutarate secretion capability enrichment in CRC. (**A)** Bar plots with 95%-confidence intervals for the share of microbiome models with the capability to produce glutarate across the sexes and cases and controls. (**B)** Bar plots with 95%-confidence intervals for the share of microbiome models with the capability to produce glutarate across different stages of colorectal cancer. Late-stage colorectal cancer had significantly higher shares of microbiomes with the capability to produce glutarate. **(C)** Statistics for the detected *Fusobacterium* species. *P*-values are from logistic regression adjusted for age, sex and BMI except for *F. necrophorum* and *F. gonidiaformans*, where *p*-values were calculated from Fisher’s exact tests due to small case numbers. (**D)** Lysine to butyrate pathway through glutarate in *Fusobacterium* spp. Note that only *Fusobacterium* spp. had the complete pathway including a exchange reaction for glutarate. CRC = colorectal cancer, MP = multiple polyps, HS = healthy after surgery

Next, we fit logistic regressions to investigate whether individual metabolite secretion capabilities are enriched in CRC microbiomes, including age, sex, and body mass index (BMI) as covariates into the regression analyses (Table S1 for full results). After correction for multiple testing, only the glutarate secretion capability remained significant and was clearly enriched in CRC cases (odds ratio (OR) = 2.51, 95% confidence interval (CI) = (1.80;3.51), *p* = 6.45e-08, FDR<0.05) (Figure 2a). Importantly, the capability to secrete glutarate was associated with the stage of disease (*p* = .003, Figure 2b), indicating that glutarate secretion potential may be an *in silico* biomarker for CRC progression, although this result was not significant after correcting for multiple testing (FDR = 0.13). Testing the association with basic covariates, we found that the glutarate production capability was enriched in men (OR = 1.64, 95%-CI:(1.17;2.29), *p* = .004) (Figure 2a), but not associated with age or BMI. To link the change in metabolic functions back to patterns of species abundance, we applied the concepts of necessity and sufficiency (see Methods). We identified 59 species fulfilling the criteria of being sufficient, meaning that all communities containing at least one of these species were able to secrete glutarate. Of these 59 species, only seven species were strictly sufficient. Strikingly, all strictly sufficient species belonged to the genus *Fusobacterium*. Importantly, of the seven *Fusobacterium* spp., two were significantly more often detected in CRC cases (Figure 2c). Together, these seven species were also necessary, meaning that at least one of the seven detected *Fusobacterium* species had to be present in the community for net glutarate production capacity. Hence, a community had a positive net production capacity for glutarate, if and only if *Fusobacterium* species were present.

Next, we aimed to identify specific network properties of *Fusobacterium* species allowing for net glutarate production capabilities. Using the AGORA resource, we found that *Fusobacterium* spp. are the only species with a complete pathway from lysine to glutarate *and* an exchange reaction for glutarate (Figure 2d). Notably, the pathway of glutarate production from lysine co-occurs with the pathway of butyrate production from glutarate^37^ (Figure 2d). Consequently, CRC microbiomes were enriched for the lysine to butyrate fermentation pathway through glutarate. In conclusion, while *Fusobacterium* spp., especially *F. nucleatum*, have been repeatedly linked to CRC, we identified a metabolic capability unique to *Fusobacterium* spp.

### *CRC microbiomes show lowered SFCA production capacities mediated by* Fusobacterium *sp. presence*

**Figure 3.**
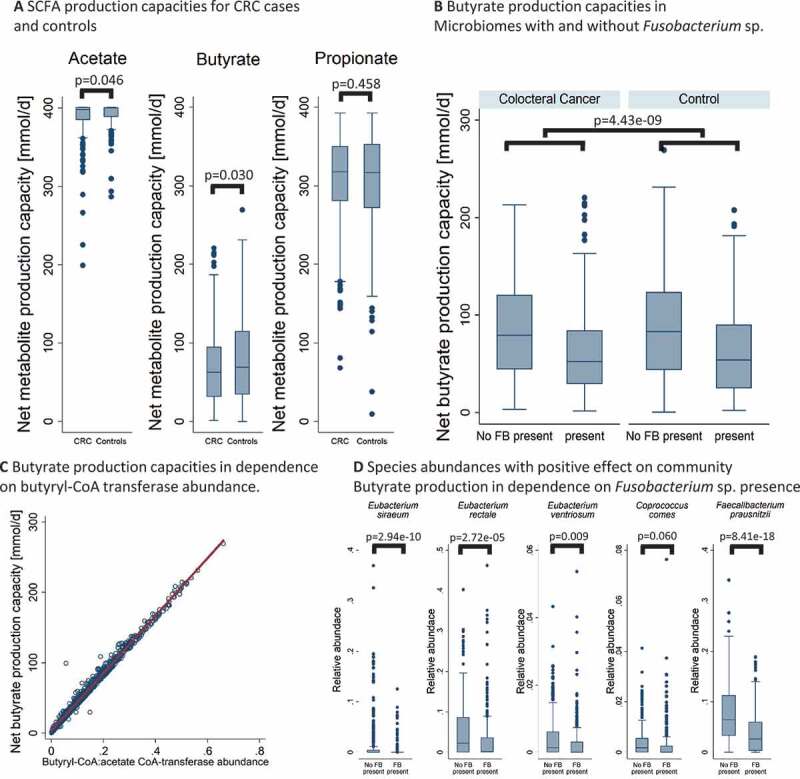
Overview over simulation results regarding short chain fatty acid production. **(A)** Box plots for acetate, butyrate, and propionate net production capacities for CRC cases and controls. Net production capacities are significantly different across cases and controls for acetate (*p* = .046) and butyrate (*p* = .030). (**B)** Box plots for net butyrate production capacities for cases and controls across microbiomes with and without *Fusobacterium* sp. presence. Communities with *Fusobacterium* sp. had significantly lower net butyrate production potentials (*p* = 4.43e-09). (**C)** Scatter plot with regression line for net butyrate production capacities in dependence on the butyryl-CoA:acetate CoA-transferase abundance (R-squared = 0.99). (**D)** Box plots for species abundances positively associated with community butyrate production in dependence of *Fusobacterium* sp. presence. SCFAs = short chain fatty acids, FB = Fusobacteria. CRC = colorectal cancer

As glutarate is an upstream metabolite of acetate and butyrate,^37,38^ we calculated the net secretion potential for SFCAs, including propionate, by community modeling and tested for differences in community secretion potentials between CRC cases and healthy controls. Strikingly, acetate (regression coefficient b = 2.88, 95%-CI:(0.05;5.71), *p* = .046) and butyrate (b = 8.98, 95%-CI:(0.87;17.10), *p* = .030) production potential but not propionate production potential (b = −3.61, 95%-CI:(−13.16;5.94), *p* = .458) was higher in healthy controls (Figure 3a). Notably, microbiomes with *Fusobacterium* sp. had lower butyrate production potential (b = −23.71, 95%-CI:(−31.52;-15.89), *p* = 4.43e-09) in cases as well as in controls (Figure 3b). No effect of *Fusobacterium* sp. presence on acetate production capacities could be identified, while propionate production potentials were higher in microbiomes with *Fusobacterium* sp. (Fig. S1). Importantly, the presence of *Fusobacterium* spp. statistically mediated the effect of CRC on butyrate production potential (Sobel-Goodman test: Indirect effect b = 5.29, 95%-CI:(2.77;7.81), *p* = 3.79e–05). Thus, our analyses provide evidence that the presence of *Fusobacterium* sp. may be deleterious for community butyrate production potential, leading to CRC microbiomes that are enriched in *Fusobacterium* spp. and have reduced butyrate production potentials.

### *Fusobacterium* sp. have large negative ecological effect on butyrate production through the butyryl-CoA:acetate CoA-transferase route

**Figure 4.**
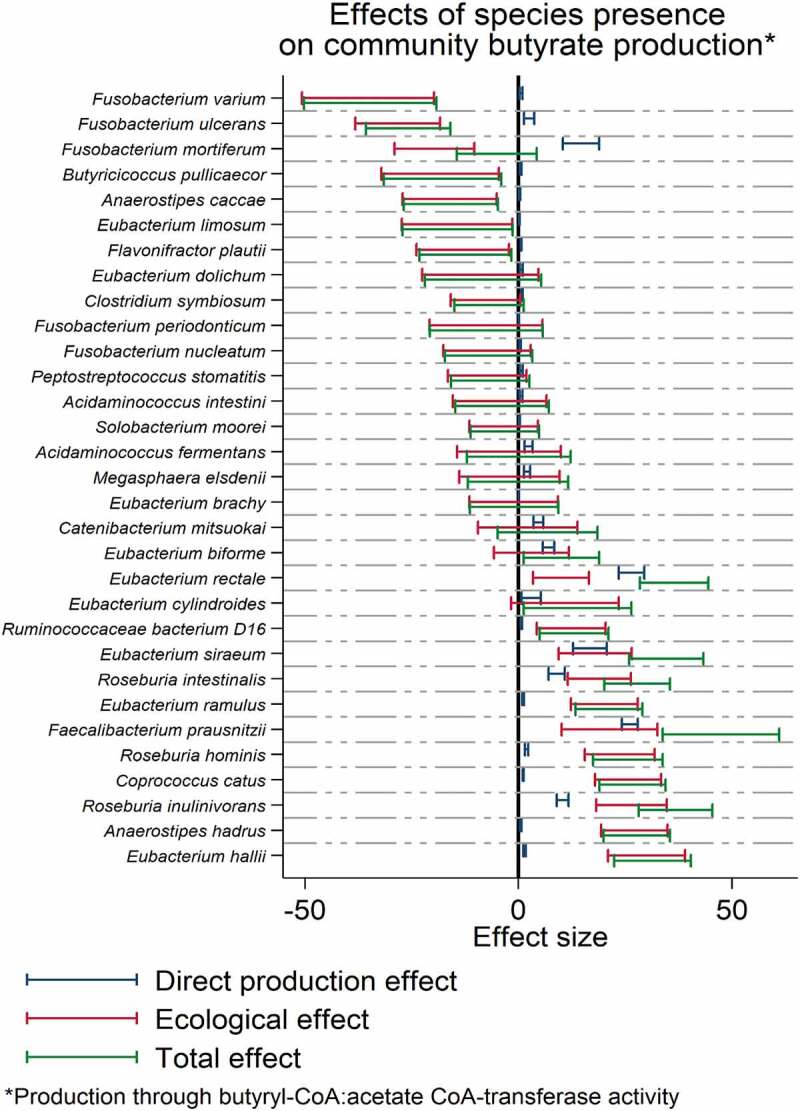
Direct, ecological and total effects of species presence on community butyrate production capacity through the butyryl-CoA:acetate CoA-transferase routs. Caps represent 95% confidence intervals. Only species found in at least 5% of all samples were included

To elucidate the changes in the community associated with *Fusobacterium* sp. that cause lower butyrate production potential, we calculated for each butyrate-producing species found in at least 5% and maximally in 95% of all samples the direct butyrate production capacity and their ecological effects on the community butyrate production (Methods). Three reaction abundances showed a correlation of r > 0.99 with the community butyrate production capacity: the conversion reaction of crotonoyl-CoA to butyryl-CoA by the Bcd-Etf complex (VMH identifier: BTCOADH), butyryl-CoA:acetate CoA-transferase (VMH identifier: BTCOAACCOAT), and ferredoxin:NAD oxidoreductase (VMH identifier: FDNADOX_H). Of these three, which belong to the same pathway, the butyryl-CoA:acetate CoA-transferase reaction directly produces butyrate with variance in its abundance being responsible for over 98% of the variance in net community butyrate production capacity. Thus, the abundance of this reaction directly translates into net butyrate production capacity in a proportional manner (R-Squared = 0.99 Figure 3c), thereby representing the main route for microbial butyrate production in the population of interrogated community models. While all five *Fusobacterium* spp. detected in at least five percent of the samples were predicted to produce small amounts of butyrate via the butyryl-CoA:acetate CoA-transferase route, they had large negative ecological effects on community butyrate production (Figure 4, Table S2). Note that while the direct species production was calculated from the individual community model within the COBRA framework, the ecological effects and total effects were explicitly defined on the population of COBRA community models and could therefore not be extracted without applying population statistics. *F. varium, F. mortiferum*, and *F. ulcerans* had the highest negative impact on community butyrate production across all modeled butyrate-producing species (Figure 4). Highlighting the negative impact of *Fusobacterium* sp. presence, among seven species that contributed at least 10% of the variance to the net community butyrate production capacity with a positive effect sign (Table S3), five were negatively correlated with the presence of *Fusobacterium* sp., although the effect regarding *Coprococcus comes* was not significant after adjusting for the study group variable (OR = 0.70; 95%-CI:(0.48;1.02), *p* = .06, Table S4). The effect was most drastic with the well-known fiber degrader *Faecalibacterium prausnitzii* (OR = 0.49, 95%-CI:(0.42;0.58), *p* = 8.41e-18, FDR<0.05 Figure 3e, Table S4), which is known to produce butyrate through the butyryl-CoA:acetate CoA-transferase route.^13^

### Fecal metabolomics validates community butyrate production predictions

**Figure 5.**
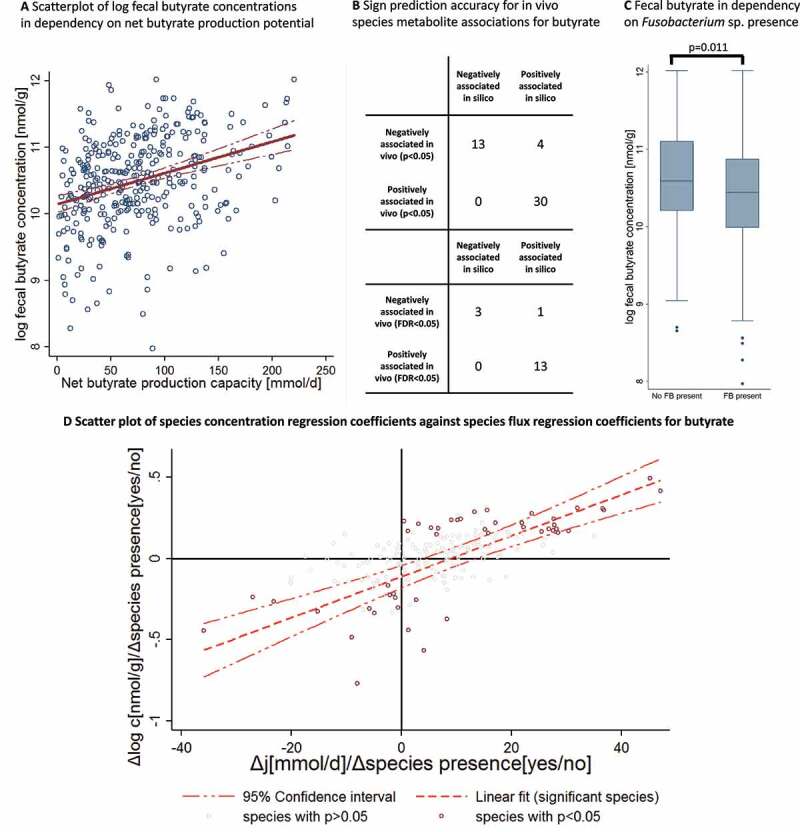
Validation of community modeling predictions regarding butyrate. **(A)** Scatter plot with regression line of log fecal butyrate concentrations against community net butyrate production capacities. The regression slope is significantly different from zero (b = 0.00445, 95%-CI:(.00295,.00595), *p* = 1.22E-08). (**B)** Accuracy of sign prediction for significant species fecal butyrate concentration association through community modeling. (**C)** Box plots for log fecal butyrate concentrations for microbiomes with and without *Fusobacterium* sp. Microbiomes with *Fusobacterium* sp. were associated with lower fecal butyrate levels (b = −0.19, 95%-C:(−0.34, −0.05),*p* = .011). (**D)** Scatter plot with regression line of empirical species fecal butyrate association statistics (expressed as regression coefficients) against *in silico* species net metabolite production association statistics (expressed as regression coefficients). *In silico* and empirical association statistics were significantly correlated with each other (r = 0.75, *p* = 9.96e-10). FB = *Fusobacterium* sp., c = concentration, j = net production capacity flux

All the results were thus far based on *in silico* calculations. Now, we focus on the validation of the core results using fecal metabolome data from the same cohort, where for 347 individuals, fecal metabolome measurements were available, including quantifications for butyrate and glutarate.^16^ The community models made distinct predictions (i) for the net butyrate production capacity, (ii) for the species contributing to community butyrate production, and (iii) for the prediction that butyrate community production is lowered in communities with prevalent *Fusobacterium* sp. First, the predicted butyrate secretion capacities were significantly correlated with the measured log fecal butyrate concentrations (b = 0.005, 95%-CI:(0.003,0.006), *p* = 9.87E-10), explaining 10.9% of fecal butyrate concentration variance overall (see Figure 5a). Second, we calculated the full species butyrate association pattern by regressing the fecal log butyrate concentrations on the species presence in sequential regressions while adjusting for case-control status, age, sex, and BMI. The corresponding *in silico* species-metabolite association statistics were then derived from analogous regressions using the net community butyrate production capacity as the response variable. The summary statistics for the species butyrate association patterns *in vivo* and *in silico* can be found in the supplementary material (Table S5). Of 47 nominally significant species fecal butyrate associations, community modeling predicted the sign correctly for 43 associations (prediction accuracy: 91.49%, Fisher’s exact test: *p* = 1.69e-08). Of 17 FDR-corrected significant species fecal butyrate associations, community modeling predicted the sign in all but one case (*Granuticatella adiacens*) (prediction accuracy: 94.1%, Fisher’s exact test: *p* = .006) (Figure 5b). Beyond the sign, community modeling predictions were additionally significantly correlated with the size of the regression-based association statistics for the nominally significant species (r = 0.75, *p* = 9.96e-10) and the FDR-corrected significant species (r = 0.86, *p* = 7.65e-06) (Figure 5d). Moreover, as predicted by the modeling, individuals with prevalent *Fusobacterium* sp. had significantly lower fecal butyrate levels (b = −0.19, 95%-C:(−0.34, −0.05), *p* = .011) (see Figure 5c) despite *Fusobacterium* spp. themselves being butyrate producers, reflecting the predicted deleterious effects of *Fusobacterium* spp. on other butyrate producing species. Importantly, this result demonstrates that the direct species effect (positive in the case of *Fusobacterium* sp.) was not sufficient to predict the empirical species-metabolite association (negative in the case of *Fusobacterium* sp.). Consequently, analyzing COBRA models at the population level increased the predictive ability with respect to metabolite-microbiome relations, transcending approaches based on analyzing individual COBRA community models.

However, with respect to the demonstrated predictive abilities, the variance in the fecal metabolome is also determined by variance in nutritional habits and attributes of the host, neither of which was modeled in this work. Therefore, the extent to which the variance in the fecal metabolome could be explained by community modeling was limited. Note that at no point was the metabolome data utilized in the construction of the COBRA community models. In conclusion, our frameworks based on COBRA community modeling were able to predict measured species butyrate correlations with high accuracy and, thus, predict the species-level contribution to the fecal butyrate pool.

### Fecal glutarate levels indicate net glutarate consumption by microbial communities

**Figure 6.**
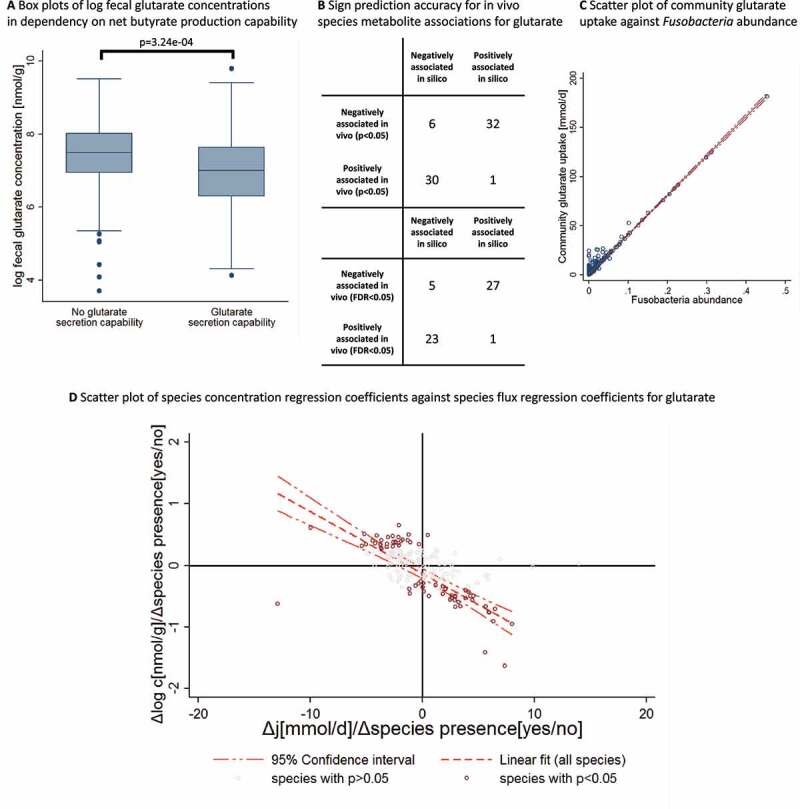
Validation of community modeling predictions regarding glutarate. (**A)** Box plots for log fecal glutarate concentrations for communities with and without glutarate secretion capability. Communities with glutarate secretion capability are associated with significantly lower fecal glutarate concentrations (b = −0.44, 95%-CI(−0.68, −0.20), *p* = 3.24e-04). (**B)** Accuracy of sign prediction for significant species fecal glutarate concentration association through community modeling. (**C)** Scatter plot of *in silico* community uptake of glutarate against *Fusobacterium* sp. abundance (r = 0.98). (**D)** Scatter plot with regression line of empirical species fecal glutarate association statistics (expressed as regression coefficients) against *in silico* species net metabolite production association statistics (expressed as regression coefficients). *In silico* and empirical association statistics were significantly correlated with each other (r = −0.76, *p* = 2.89e-14). c = concentration, j = net production capacity flux

Then, we turned our attention to the relation between *in silico* predicted net glutarate production capacity and actual experimentally measured fecal glutarate concentrations. Surprisingly, we discovered that communities with the capability of glutarate production were associated with significantly lower glutarate levels in feces (b = −0.44,95%-CI(−0.68, −0.20), *p* = 3.24e-04) (see Figure 6a), explaining 4.06% of the variance in fecal glutarate pools. Consequently, fecal glutarate concentrations were significantly lower in the presence of *Fusobacterium* sp. As shown above, glutarate production capability is the consequence of *Fusobacterium* sp. presence, meaning that glutarate production capability was associated with reduced fecal glutarate concentrations as well. The microbial transport reaction for glutarate is bi-directional and the necessary reactions of glutarate production co-occur with the degradation reactions leading to butyrate production from glutarate (Figure 2d). Hence, it is possible that a positive net glutarate production capacity indicates that glutarate can be taken up for ATP generation. In this scenario, communities would be able to consume glutarate, explaining the inverse association of net metabolite production capacity and fecal metabolite concentration. This interpretation was corroborated by testing the ability of community modeling to predict species fecal glutarate associations (Table S6). Among 69 nominally significant species fecal glutarate associations, 62 were in line with the community modeling prediction when interpreting the secretion potential as a measure of consumption (prediction accuracy: 89.86%, Fisher’s exact test: *p* = 2.28e-12) (Figure 6b). For 50 out of 56 FDR-corrected significant associations, community prediction correctly predicted the sign (prediction accuracy: 89.39%, Fisher’s exact test: *p* = 1.27e-09) (Figure 6b). As with butyrate, community modeling was also able to predict the size of the regression coefficient of the species for the fecal glutarate concentration (r = −0.76, *p* = 2.89e-14 for the nominally significant species; r = −0.74, *p* = 5.36e-11 for the FDR-corrected species) (Figure 6d).

### *Fecal glutarate consumption is driven by* Fusobacterium *spp*. in silico

Above, we showed that community glutarate secretion *in silico* is likely an indicator of glutarate consumption *in vivo*. To test this interpretation, we designed additional simulations to model the species that were capable of consuming glutarate. Note that while only *Fusobacterium* spp. were able to secrete glutarate, we identified 16 species present in at least one microbiome that were able to take up glutarate, including the seven detected *Fusobacterium* spp. (Table S7). However, *Fusobacteria* abundance was the primary determinant of glutarate uptake potential (R-squared = 0.97, see Figure 6c). Consequently, the uptake potential and the community secretion potential for glutarate correlated strongly with each other (r = 0.98, *p* < 1e-30, Figure 6c). In conclusion, the interpretation of the community glutarate production capacity as an indicator of the potential to consume glutarate was also supported by the species-level uptake modeling.

## Discussion

A key challenge in obtaining a mechanistic understanding of the gut microbiome in health and disease is to map changes in gut microbial abundances onto functional changes impacting the host’s metabolism. With recent developments allowing for the personalization of COBRA community models of gut microbial metabolism,^34^ new possibilities have emerged to translate COBRA predictions for metabolic functions to the population level.^35^ Instead of interrogating a single computational metabolic model, attributes of computational metabolic models can now be investigated across strata of individuals by integrating available metadata, such as age, sex, and disease status.^34^ However, classical modeling strategies, such as COBRA modeling, ODE modeling,^25^ and agent-based modeling,^39^ are designed to deliver insights into the *concrete* system under investigation.^40^ In themselves, those computational methods do not make use of the information provided by the variance in the microbiome community composition in conjunction with metadata across a population of models in a systematic way. Therefore, tailored population statistics approaches are needed to capitalize both on the biological information encoded in computational models and on the variance in microbiome composition at the population level.

Here, we present a functional metabolic modeling approach combining COBRA modeling with population statistics that not only translates individual-specific microbial abundances into personalized microbial metabolite profiles, but also investigates these profiles at the population level. The theoretical concepts developed in this study therefore pave the road for the application of COBRA microbial community modeling to large cohorts with microbiome measurements. To demonstrate its utility, we applied the methodology herein introduced to a large public metagenomic dataset from Yachida et al.^16^ and investigated the metabolic functions of the microbiome in the context of CRC.

By applying this framework to CRC data, we demonstrated that each person’s gut microbiome seems to be functionally unique, emphasizing the need for individualized modeling of microbiomes as possible with COBRA microbial community modeling. We highlight the utility of our approach by generating insights into the functional alterations associated with *Fusobacterium* sp. presence in the gut microbiome and insights of potential clinical relevance especially in CRC where *Fusobacterium* spp. are enriched.^41–43^ Finally, we validated the prediction of *in silico* modeling against fecal metabolome data, revealing excellent agreement between *in silico* predictions and empirical data.

The analyses of net production capacities revealed alterations in the domain of fermentation products in CRC, including SFCAs. CRC microbial communities had lower net production capacities in acetate and butyrate (Figure 3). The lower production capacity of SFCAs is of potential clinical relevance due to the known anti-inflammatory, antitumor effects of butyrate.^7^ Moreover, butyrate is a main energy source for colonocytes but not for cancer cells, which prefer glucose.^7^ Evidence exists for butyrate having protective properties for colon-cells, and low fiber intake has been considered to be a risk factor for CRC.^14^ The finding that CRC microbiomes have decreased capacities to produce butyrate agrees with earlier observations of depletion of butyrate producing species in CRC microbiomes.^44,45^

Although well documented, the cause for the depletion of butyrate producing species in CRC is less understood. Here, we found that the presence of *Fusobacterium* sp. is strongly associated with this shift in community composition, which was quantified by the highly negative ecological effect of *Fusobacterium* sp. on community butyrate production (Figure 3). Importantly, the negative effect of *Fusobacterium* sp. is not a CRC-specific feature. In healthy individuals, the presence of *Fusobacterium* sp. was associated with lower butyrate production capacities accordingly (Figure 3b). This observation fits well with *in vitro* studies showing that *F. nucleatum* produces bactericidal compounds hazardous to butyrate-producing species, in this case *F. prausnitzii*.^46^ Of note, the highest negative effects on community butyrate production were with *F. varium, F. mortiferum*, and *F. ulcerans*, indicating that not only *F. nucleatum* may play a role in CRC (Figure 4). *Fusobacterium* spp. co-occur with each other,^43^ making inferences about a single species complicated. For example, in the present study, we also found *F. mortiferum* to be significantly enriched in CRC (Figure 2c). In conclusion, the evidence points overall toward *Fusobacterium* spp. being deleterious for community butyrate production.

*F. nucleatum* has been repeatedly implicated in CRC.^47–49^ While it has been described that *F. nucleatum* plays a role in treatment resistance in CRC and in the modulation of antitumor inflammatatory response,^50,51^ the metabolic role of an enrichment in *F. nucleatum* and other *Fusobacterium* spp. in CRC is less clear. In this respect, we found clear enrichment of the capability to produce glutarate from lysine in CRC microbiomes, which is mechanistically linked to *Fusobacterium* sp. presence (Figure 2d). Importantly, this feature is a metabolic trait of all seven *Fusobacterium* spp. detected in this study and a general feature of all species in the *Fusobacterium* genus captured in the VMH resource.^36^ *Fusobacterium* spp. are the only species in the AGORA collection having the full pathway from lysine to glutarate *and* an exchange reaction for glutarate. In line with this study, *Fusobacterium* spp. are known for asaccharolytic metabolism.^52^ As glutarate is an intermediate in the pathways from lysine and from glutarate to butyrate,^37^ this suggests that the increased *Fusobacterium* abundance in CRC microbiomes would result in increased amino acid fermentation, in particular lysine to butyrate. Importantly, as *Fusobacterium* spp. are associated with a lower abundance of species producing butyrate from carbohydrates, such as *F. prausnitzii*, the overall effect of *Fusobacterium* spp. on community butyrate production is negative. An enrichment in amino acid degradation pathways accompanied by a corresponding decrease in carbohydrate degradation has been reported for CRC microbiomes,^53^ consistent with our results. It is noteworthy that we found *Fusobacterium* sp. to be enriched in men. Men have higher risks for developing CRC,^54^ sparking the speculation of whether *Fusobacterium* sp. presence may mediate a part of the sex-specific risk for CRC, although the discussion around sex-differences in CRC is complicated by social and cultural effects.^55^

Of note, the case of *Fusobacterium* spp. highlights the necessity of statistical approaches when utilizing COBRA metabolic modeling at the population level. Additional to direct contributions to net secretion, which could be directly calculated from COBRA modeling approaches without using population statistics, microbes can also influence the composition of microbial communities, changing community metabolite secretion by ecological processes. These ecological effects, coded in the multivariate abundance patterns across microbial communities, are not directly quantifiable from COBRA modeling alone. By integrating information on compositional variance across all available models, our methodology enables the calculation of ecological effects. In return, the negative effects of *Fusobacterium* spp. on community butyrate production, which are reflected in the fecal metabolome data, become visible. Moreover, following this approach, we achieved a high accuracy across the microbiome in terms of predicting *in vivo* butyrate-species associations. This example demonstrates that the accuracy of predictions from COBRA metabolic modeling can be increased through population statistics.

Previously, we demonstrated the use of personalized metabolic modeling for the stratification of pediatric inflammatory bowel disease patients and controls in a *purely in silico* approach^32^ and validated changes in the metabolome of Parkinson’s disease patients with personalized models built from an unrelated cohort.^35^ Here, by integrating the AGORA-based COBRA microbial community modeling predictions with fecal metabolomics, we validated *in silico* predictions regarding butyrate, glutarate, *Fusobacterium* sp. and other butyrate-producing species. We were able to correctly predict, which species correlated with fecal butyrate and glutarate levels, and even the effect sizes of these associations were predicted correctly to a high degree (Figures 4 and 5). This functional metabolic modeling approach that integrates population statistics methods delivers a new proof of principle for community modeling, opening new routes for applications. As butyrate production is considered to be integral for gastrointestinal health,^14^ probiotic, prebiotic, and synbiotic interventions have started targeting beneficial butyrate producers, such as *F. prausnitzii*.^56^ AGORA-based community modeling enables the prediction of the outcomes of therapeutic and dietary interventions.^57^ Our study now reveals that these *in silico* biomarkers are indeed reflective of the gut microbiome’s metabolic capacities and are in good agreement with fecal butyrate concentrations. Importantly, the models were not contextualized with the metabolome data from Yachida et al. during their construction, meaning that the Yachida et al. dataset delivers an external validation.^16^ Thus, *in silico* modeling can deliver computational biomarkers for phenotypes, which could be used, in principle, for diagnostic or prognostic purposes. Additionally, the presented work highlights that community modeling can be utilized as a further layer of validation for empirical species metabolome association studies where correlations are often difficult to interpret due to uncontrolled confounding.^21^ As community modeling is based on deterministic calculations from microbiome measurements, certain types of confounding have no effect on *in silico* species metabolite association. Therefore, community modeling can diminish false positives in microbiome metabolome association studies; an important aspect as noted in earlier work.^21^

While the modeling was overall in good agreement with the empirical metabolome measurement, several limitations should be noted. We applied one standardized diet, therefore excluding variance caused by differential dietary habits from the analyses. However, the general methodology allows the personalization of the diet information used for modeling. Thus, if dietary habits are sampled in a suitable way, the type of calculation performed here can be individualized regarding microbial abundances and based on diet information.^33^ Furthermore, this study did not integrate host metabolism into the modeling. Based on whole body organ resolved COBRA modeling,^30^ further studies could deliver more insight into the interplay between the host and the microbiome in CRC and beyond. Knowledge about microbial functions and genomic annotations is incomplete, and as such, the AGORA collection is subject to constant updates. Another known limitation of COBRA is the lack of kinetic parameters and the simulation of fluxes rather than concentrations due to the steady-state assumption. However, the good agreement between *in silico* fluxes and experimentally measured concentrations in this study suggests that it is possible to mechanistically translate increased or decreased fluxes into increased or decreased concentrations. Importantly, this study is based on cross-sectional data, and as such, causality between clinical parameters and microbial functions cannot be established. However, community modeling for determining metabolic function cannot be confounded by factors, such as age, sex, exercise, or other factors, as they are deterministic calculations from abundance patterns. By providing a major conceptual advantage regarding the functional analyses of species metabolite associations by calculating abundance concentration correlations, community modeling allows for the dissection of direct contributions of species to and their ecological effects on the community metabolite production capacities. Notably, ecological effects, as defined in this work, allow the mapping of the statistical effects of the presence of species on the community structure in terms of metabolic function. Ecological effects are notions of statistical associations, however. Therefore, they cannot be interpreted in the sense of causal statistics^58^ without further theoretical considerations.

In conclusion, AGORA-based community modeling provides a powerful toolset for the characterization of microbial metabolic functions in health and disease, delivering testable hypotheses, *in silico* biomarkers, and potential endpoints for clinical studies. Importantly, AGORA reconstructions have been extensively curated based on comparative genomics and experimental data from two microbial textbooks and over 200 peer-reviewed papers.^23^ Thus, beneath the conclusions presented in this paper lies accurate, manually gathered knowledge on fermentation pathways in hundreds of organisms. Overall, this study provides a proof of principle that the knowledge encoded in AGORA models can be translated into clinical insight via community modeling.

## Patients and methods

### Study sample

We utilized the Japanese colorectal cancer cohort data from the worky by Yachida et al.,^16^ which had publicly available shotgun sequencing data for n = 616 individuals (365 CRC cases and 251 healthy controls). The reads had already been processed and taxonomic profiling utilizing MetaPhlAn2 was performed.^59^ Attached to this dataset several, meta-data on age, sex, BMI, smoking, alcohol, stages of the disease, and tumor location were available. Additionally, linked to these data, fecal metabolome quantifications were available for n = 347 probands (CRC: 220, controls: 127), allowing the validation of attributes of the community models by linking them to empirical metabolome quantifications. For details on metagenomic and metabolomic measurements, refer to Yachida et al..^16^

### Definition of an average Japanese diet

An average Japanese diet was defined based on the mean daily food consumption in 106 Japanese extracted from food frequency questionnaires and 28 days weighed diet records^60^ (Table S8a). Therefore, we used the Diet Designer of the VMH database (https://vmh.life), which lists the composition of >8,000 food items.^36^ In the absence of a perfect match, the most related food item entries were retrieved. The Diet Designer calculates uptake flux values in mmol/person/day for each nutrient component based on the specified diet, as described elsewhere.^36^ We integrated these uptake flux values as diet constraints with all community microbiome models using the Microbiome Modeling Toolbox^33^ (see below). To ensure that all AGORA pan-species models could grow under the defined diet, we adapted the calculated uptake fluxes as necessary (Table S8b). The diet constraints were defined to be in mmol/person/day.

### Simulations

All simulations were performed in MATLAB (Mathworks, Inc.) version R2018b with IBM CPLEX (IBM) as the linear and quadratic programming solver. The simulations relied on functions implemented in the COBRA Toolbox,^26^ and the Microbiome Modeling Toolbox.^33^

### Construction of sample-specific gut microbiota models

Metagenomic datasets from 616 samples were used as published in.^16^ We utilized the sequencing data from the corresponding supplementary material (https://static-content.springer.com/esm/art%3A10.1038%2Fs41591-019-0458-7/MediaObjects/41591_2019_458_MOESM3_ESM.xlsx). The data had been already preprocessed and was available in relative abundances on the species level. The relative abundances were mapped onto the reference set of 773 AGORA genomes^23^ through the translateMetagenomeToAGORA.m function in the Microbiome Modeling Toolbox.^33^ Via the mgPipe module of the Microbiome Modeling Toolbox, personalized microbiome models were derived. In brief, the corresponding AGORA reconstructions of all strains found in at least one microbiome were put together into one global constraint-based microbiome community reconstruction as described before.^33,61^ Then, the biomass objective function was coupled with the flux through each AGORA species pan-model (for details see^62^), parametrizing the community biomass reaction via the relative abundances as stoichiometric values for each microbe biomass reaction in the community biomass reaction. The models were appropriately contextualized with the average Japanese Diet described above. Next, the resulting diet exchange fluxes were applied to community models.^33^ The flux through the community biomass reaction was set to be between 0.4 and 1 mmol/person/day, as described before. The features of the personalized community models are given in Table 1.

## Definitions and theoretical frameworks

### Utilized attributes of populations of community models

Let $M=\left\{M_{1},M_{2},\ldots M_{I}\right\}$ be a set of *I* community models corresponding to *I* measured microbiomes. We are interested here in three attributes of the model $M_{i}$:

- the vector of microbial abundances $a_{i}\in {\left[0,1\right]}^{K}$ belonging to the model $M_{i}$ where *K* denotes the number of species included into the AGORA collection.
- the vector of reaction abundances $r_{i}\in {\left[0,1\right]}^{J}$belonging to the model $M_{i}$ where *J* denotes the number of unique reactions included into the AGORA collection in total.
- the vector of net metabolite production capacities $n_{i}\in {\left[0,c_{l}\right]}^{L}$ with $c_{l}$ being the maximum possible net metabolite production capacity under the set of applied constraints and *L* being the number of metabolites with microbial exchange reactions in at least one AGORA genome-scale model. Net metabolite production capacities are defined by the absolute value of the difference between the maximal flux through the secretion exchange reaction and the minimal flux through the corresponding dietary uptake exchange reaction. $n_{il}>0$.

Thus, our population statistics analyses of community models were performed on microbial abundances, reaction abundances, net metabolite production capacities and net metabolite production capabilities.

### Metabolic equivalence

Now, we define the term metabolic equivalence, which allows us to cluster microbial communities having the same set of metabolic functions.

***Definition 1: Metabolic Equivalence***

We call two community models $M_{j}$ and $M_{k}$ metabolic equivalent regarding the (sub)set E of metabolites with exchange reactions in at least one AGORA genome scale model if and only if $for\;\;all\;\;l\in E\;\;it\;\;holds\;\;that\;\;n_{jl}>0\Leftrightarrow n_{kl}>0$. We then write $M_{j}{∼}_{E}M_{k}$.

This defines an equivalence relation, as the relation ${∼}_{E}$ fulfills the attributes of being reflexive ($M_{j}{∼}_{E}M_{j}$), symmetric ($M_{j}{∼}_{E}M_{k}\Leftrightarrow$ $M_{k}{∼}_{E}M_{j}$), and transitive ($M_{j}{∼}_{E}M_{k}\;\;and$ $M_{k}{∼}_{E}M_{l}\Rightarrow M_{j}{∼}_{E}M_{l}$).

### Necessary and sufficient conditions for net metabolite production capacities

Now, we define *sufficient* and *necessary* attributes for net metabolite production capabilities given a set of microbial community models $M=\left\{M_{1},M_{2},\ldots M_{I}\right\}$. The concepts of *“metabolically sufficient”* and *“metabolically necessary”* will be analogous for species and reactions. First, however, we will define *informative metabolites*.

***Definition 2: Informative metabolite***

We call a metabolite l informative, if and only if $\exists \;\;M_{i}\in M:n_{il}>0\;\;and\;\;\exists \;\;M_{j}\in M:n_{jl}=0.$ Informative metabolites are therefore those metabolites with variance in the net production capabilities across the set of models *M*.

***Definition 3: Necessary and sufficient reactions***

Let l be an informative metabolite. Then, we call a reaction *k* necessary if and only if for all $M_{i}\in M$ it holds that $r_{ik}=0\Rightarrow n_{il}=0$. We call a reaction *k* sufficient if and only if for all $M_{i}\in M$ it holds that $r_{ik}>0\Rightarrow n_{il}>0$.

***Definition 4: Necessary and sufficient species***

Let l be an informative metabolite. Then, we call a species *j* necessary if and only if for all $M_{i}\in M$it holds that $a_{ij}=0\Rightarrow n_{il}=0$. We call a species *j* sufficient if and only if for all $M_{i}\in M$ it holds that $a_{ij}>0\Rightarrow n_{il}>0$.

Thus, we call species and reactions *necessary* for a certain metabolic function if their absence implies missing the metabolic function under consideration in all observed community models. In contrast, we call species and reactions *sufficient* for a metabolic function if their presence implies the presence of the metabolic function of interest in all models. It is important to note that the concepts of necessity and sufficiency are defined for metabolites, which are neither produced by all models, nor by any of the models. *Necessary* and *sufficient* conditions can only be learnt from variance in the occurrence, which motivates the definition of *informative metabolites*. This concept is mirrored in statistics, where variance in the random variables is a prerequisite to identify patterns of stochastic dependency. As it is in statistics, the dependency relations given by sufficiency and necessity should not be confused with causality, as conditions could co-occur in the set communities observed. Therefore, we define the concepts of *strictly sufficient* and *strictly necessary*, which introduces a type of conditional dependence notion.

***Definition 5: Strictly necessary reactions***

Let l be an informative metabolite. Let $Q_{l}$ be the set of all reactions, which are necessary for the net production capability for the metabolite *l* and $k\in Q_{l}$ a specific necessary reaction. We call *k* strictly necessary if and only if $\exists \;\;M_{i}\in M\;\;with\;\;r_{ik}=0\;\;and\;\;\forall \;j\in Q_{l}\setminus k:r_{ij}\neq 0$.

***Definition 6: Strictly necessary species***

Let l be an informative metabolite. Let $Q_{l}$ be the set of all species, which are necessary for net production capability for *l* and $k\in Q_{l}$ a specific necessary species. We call *k* strictly necessary if and only if $\exists \;\;M_{i}\in M\;\;with\;\;a_{ik}=0\;\;and\;\;\forall \;j\in Q_{l}\setminus k:a_{ij}\neq 0$.

***Definition 7: Strictly sufficient reactions***

Let l be an informative metabolite. Let $Q_{l}$ be the set of all reactions, which are sufficient for net production capability for *l* and $k\in Q_{l}$ a specific sufficient reaction. We call *k* strictly sufficient if and only if $\exists \;\;M_{i}\in M\;\;with\;\;r_{ik}>0\;\;and\;\;\forall \;j\in Q_{l}\setminus k:r_{ij}=0$.

***Definition 8: Strictly sufficient species***

Let l be an informative metabolite. Let $Q_{l}$ be the set of all species, which are necessary for net production capability for *l* and $k\in Q_{l}$ a specific necessary species. We call *k* strictly necessary if and only if $\exists \;\;M_{i}\in M\;\;with\;\;a_{ik}>0\;\;and\;\;\forall \;j\in Q_{l}\setminus k:a_{ij}=0$.

It is important to realize that the definitions presented here are dependent on the variance structures in the population of microbial communities. The larger the sample size, the more necessary and sufficient conditions will be discovered. Sufficiency and necessity are technical attributes of populations of community models in the first place. The identified conditions do not need to be necessary and sufficient *in a biological sense*. However, they are valuable candidates for being indicators of causal processes and, hence, plausible targets for experimental validation.

### Direct, ecological, and total effect of species on net community metabolite production capacities

Here, we define formally the effects of a presence of a species on the net community metabolite production capacities observed in a population of community models *M*. The concepts of effects are defined via populations statistics. Therefore, these concepts must be treated as statistical estimates and should always be reported with confidence intervals. Importantly, the application of these concepts is only sensible for secretion fluxes, whose distributions are not truncated by the constraint settings (e.g., accumulations of observations at the upper bound of the possible flux range).

***Definition 9: Average direct species net production effect***

Let *l* be a metabolite and *M* the population of community models. Let $M^{j}:\{M_{i}:a_{ij}>0\}$ be the set of community models, where the abundance of the species *j* is greater than zero. The average direct species production effect ${\overset{ˉ}{d}}_{lj}$ for a metabolite *l* and a species *j* is defined by

(1) $${\overset{ˉ}{d}}_{lj}:=\frac{1}{\left|M^{j}\right|}\underset{M_{i}\in M^{j}}{\sum }d_{ilj}$$

where $d_{ilj}$ stands for the net production (through secretion and uptake) of the metabolite *l* by the species *j* in the community model $M_{i}$. We call $d_{ilj}$ the direct species net production capacity.

A species, however, cannot only impact the net community production capacity by direct contributions. A species can also impact the production of other microbes and can be associated with alteration in the community structure, changing the abundance of other microbes relevant for the community production of a metabolite. These processes motivate the definition of the *ecological species effect*, which gives a measure of these indirect influences associated with the presence of a microbe.

***Definition 10: Ecological species effect***

Let *l* be a metabolite and *M* the population of community models. Let $M^{j}:\{M_{i}:a_{ij}>0\}$ be the set of community models with the abundance of the species *j* greater than zero, and $M^{\neg j}:\left\{M_{i}:a_{ij}=0\right\}$ the set of community models missing the species *j*. The ecological species effect ${\overset{ˉ}{e}}_{lj}$ is then given by

(2) $${\overset{ˉ}{e}}_{lj}:=\frac{1}{\left|M^{j}\right|}\underset{M_{i}\in M^{j}}{\sum }\left(n_{il}-d_{ilj}\right)\;-\frac{1}{\left|M^{\neg j}\right|}\underset{M_{i}\in M^{\neg j}}{\sum }n_{il}$$

Thus, the ecological species effect is the difference between the average net metabolite production capacities of the communities having a certain species and the communities missing this certain species after discounting the direct species net production capacity. Note that the direct species net production is zero in all models belonging to the set $M^{\neg j}$.

Obviously, the ecological species effect is not necessarily causal. However, by using multivariable regressions, it can be calculated conditional on a set of covariates, minimizing confounding by basic covariates, such as age, sex, or BMI.

***Definition 11: Total species effect***

Let *l* be a metabolite and *M* the population of community models. Then, the total species effect ${\overset{ˉ}{t}}_{lj}$ is defined by the sum of average direct species net production effect and the ecological species effect:

(3) $${\overset{ˉ}{t}}_{lj}:={\overset{ˉ}{e}}_{lj}+{\overset{ˉ}{d}}_{lj}$$

The total species effect is the difference in net production capacities between the community models having a certain species and the community models missing this specific species.

## Statistical analyses

We performed statistical analyses of the following attributes of community models: (1) net metabolite production capabilities, (2) net metabolite production capacities, (3) reaction abundances, and (4) species abundances. Due to one infeasible model, the final sample size for analyzing relations between metadata and attributes of the community models was n = 615 and the final sample size for analyzing the community models together with the fecal metabolome was n = 346. For descriptive statistics, metric variables were expressed in means and standard deviations, categorical variables were described by proportions. All *p*-values are reported two-tailed. The statistical analyses were performed with STATA 14/MP (STATA Inc., College Station, Texas, USA).

### Analyses of net metabolite production capabilities

To investigate the potential differences in net metabolite production capabilities between cases and controls, we fitted logistic regressions with the net metabolite production capability as binary response variable (can be produced vs. cannot be produced). The predictor of interest in these logistic regressions was the group variable (binary: CRC cases vs. controls) and age, sex, and BMI were used as covariates to minimize confounding. We analyzed only metabolites for which at least 5% and maximally 95% of all community models could produce those metabolites to avoid unstable statistical estimates due to low case numbers. Forty-four metabolites fulfilled this criterion. Accordingly, we corrected for multiple testing using the false discovery rate (FDR),^63^ acknowledging 44 significant tests. An FDR of 0.05 was chosen as significance threshold.

In a second series of logistic regressions, we checked for associations of net metabolite production capabilities with the CRC stage. Thus, we performed logistic regressions as before exchanging the study group variable for the stage variable (categorical: surgery, multiple polyps, stage 0, stage I/II, stage III/IV) excluding healthy controls from the analysis. The stage variable was then tested on significance using a standard Wald test.^64^ Once again, we corrected for multiple testing using the FDR, adjusting the significance threshold for 44 tests. Summary statistics for both series of logistic regressions can be found in supplementary Table S1.

Post hoc, glutarate production capability, being the main result of the screening described above, was checked on associations with basic covariates. To check for association with age and sex, a logistic regression with the net glutarate production capability as response variable was fitted using age and sex as predictors of interest, while adjusting for the study group variable (binary: CRC cases vs. controls). To check for association with BMI, a logistic regression with the net glutarate production capability as response variable was fitted using the BMI as predictor of interest, while adjusting for, age, sex, and the study group variable (binary: CRC cases vs. controls).

### Analyses of net SFCA production capacities

Next, we tested the association of CRC with net production capacities of SFCAs, namely acetate, butyrate, and propionate. To this end, we fitted linear regressions using the respective net SFCA production capacity as response variable, the study group variable (binary: CRC cases vs. controls) as predictor of interest, and age, sex, and BMI as covariates. Heteroscedastic standard errors were applied in the main analyses. For sensitivity analysis, non-parametric bootstrap-derived confidence intervals were calculated using 2000 replications, but the results remained virtually unchanged. Next, we tested net SFCA production capacities on association with the presence of *Fusobacterium* sp. Once again, we used linear regressions as before using in this iteration the presence of *Fusobacterium* sp. (binary: *Fusobacterium* sp. present vs. *Fusobacterium* sp. not present) as predictor of interest, correcting for age, sex, BMI and study group by including them as covariates. Additionally, we ran mediation analysis according to the Sobel-Goodman procedure,^65^ testing whether *Fusobacterium* sp. presence mediated the effect of CRC on net butyrate production capacities. Confidence intervals for the indirect and direct effects were calculated by bootstrapping using 2000 replications.

### Analyses of direct species production effects and ecological species production effects regarding butyrate

To calculate direct and ecological species effects regarding butyrate, we first screened all reaction abundances on correlation with the net community butyrate production capacities, finding the butyryl-CoA:acetate CoA-transferase as one of the top hits. Then, we derived for 31 species (found in at least 5% and maximally 95% of all samples), the direct species production effect, the ecological species effect, and the total species effect on net community butyrate production through the butyryl-CoA:acetate CoA-transferase. The direct species production effect was calculated by using the regression equation of the butyryl-CoA:acetate CoA-transferase net community butyrate production relation, replacing the butyryl-CoA:acetate CoA-transferase abundance by the species abundance. This is justified as butyryl-CoA:acetate CoA-transferase abundance is the sum of all species abundances having the butyryl-CoA:acetate CoA-transferase reaction. Then, the ecological species and the total species effects for the 31 species were calculated according to the equations (2) and (3). Finally, 95%-CIs were calculated for all effects, using standard procedures for estimating CIs for arithmetic means.

To illustrate the effects of *Fusobacterium* spp., we explored the effect of *Fusobacterium* sp. presence on those species, which had the highest positive effect on community butyrate production, contributing at least 10% of variance with a positive effect sign. Seven species (*Copprococcus comes, Eubacterium rectale, Eubacterium siraeum, Eubacterium ventriosum, Faecalibacterium prausnitzii, Roseburia intestinalis, and Roseburia inulinivorans*) fulfilled these criteria. Then, we fitted a series of seven fractional logistic regressions^34^ with the abundance of the seven species as response variables, the presence of *Fusobacterium* sp. (binary: present vs. not present) as predictor of interest, while adjusting for age, sex, BMI, and the study group variable. We corrected the significance level for multiple testing using the FDR, adjusting the significance levels for seven tests.

Full results and summary statistics can be found in the supplementary material (Tables S2-S4).

### Statistical integration of community modeling with fecal metabolomics

To validate the simulation results regarding glutarate and butyrate, we integrated the simulation data systematically with fecal metabolome measurements in 347 individuals of the same cohort, including quantifications of glutarate and butyrate concentrations.^16^ Note that the fecal metabolome is a representative of human metabolism, diet intake, and microbial metabolism such that it cannot be expected that the microbiome can fully explain variegation in fecal metabolite profiles. However, as the microbiome is one source of variance in fecal metabolite content and as the simulations predict systematic variance in microbiome secretion profiles across the modeled individuals, we expected that the association pattern between microbes and metabolite production capacities is reflective of the association pattern between microbes and fecal metabolite concentrations. For statistical analyses, fecal glutarate and butyrate concentrations were log-transformed, minimizing the skewness of the distributions.

First, we regressed the measured fecal butyrate and glutarate concentrations on the net community production capacities via linear regressions, including age, sex, BMI, and the study group variable as covariates. In the case of glutarate, we also included the net production capability (binary: can be produced vs. cannot be produced) into the regression model, as only 52% of all models had a net production capacity bigger zero. We evaluated the predictive value of the net community production capacity, respectively, capability by testing their regression coefficients on zero and calculating the incremental R-squared values (increase in model fit by adding net production capacity/capability variables).

Next, we calculated the full species fecal butyrate concentration association pattern by running linear regressions with the measured fecal butyrate concentration as response variable, the species presence (binary: species present vs. species not present) as predictor of interest, while including age, sex, BMI, and the study group variable as covariates. Heteroscedastic standard errors were used. These regressions were run for all species, which were detected in at least 5% and maximally 95% of all samples, resulting in 181 regressions. We retrieved the regression coefficient of the species presence, the corresponding *p*-value, and the FDR, correcting for 181 tests. In a second step, we derived the full species net community butyrate production capacity association pattern in the same way. Note that the *in silico* association pattern was derived on the full sample n = 615, assuming implicitly that fecal metabolome measurements were missing completely at random. Then, we checked for all species fecal butyrate concentration associations with *p* < .05, respectively, FDR<0.05, whether the sign of the *in silico* derived regression coefficient for the species butyrate association predicted the sign of the empirically derived *in vivo* regression coefficient via Fisher’s exact test. Moreover, we correlated the two species-butyrate association statistics with each other and tested the Pearson correlation via the standard test on significance. A significant prediction of sign and size of empirically derived regression coefficients was interpreted as a validation of the community modeling. We repeated the same methodology for glutarate.

Summary statistics for the full glutarate and butyrate association patterns, *in silico* as well as *in vivo*, can be found in the supplementary material (Table S5, S6).

## Supplementary Material

Supplemental Material
