# Supplementary material for: Integration of constraint-based modeling with fecal metabolomics reveals large deleterious effects of Fusobacterium spp. on community butyrate production
Source: Gut Microbes. 2021 May 31;13(1):1915673. doi: 10.1080/19490976.2021.1915673 (PMC8168482; doi:10.1080/19490976.2021.1915673)
Supplement: Supplemental Material [file KGMI_A_1915673_SM7194.zip › Supplementary information/Supplementary_Figure_Revision.docx]

**Integration of constraint-based modeling with fecal metabolomics reveals large deleterious effects of *Fusobacterium* spp. on community butyrate production**

Johannes Hertel^1,2^, Almut Heinken^1,3^, Filippo Martinelli^1,3^, Ines Thiele^1,3,4,5*^

^1^ School of Medicine, National University of Galway, Galway, Ireland

^2^ Department of Psychiatry and Psychotherapy, University Medicine Greifswald, Germany

^3^ Ryan Institute, National University of Galway, Galway, Ireland

^4^Discipline of Microbiology, National University of Galway, Galway, Ireland

^4^ APC Microbiome Ireland, University College Cork, Cork, Ireland

*Corresponding Author: ines.thiele@nuigalway.ie


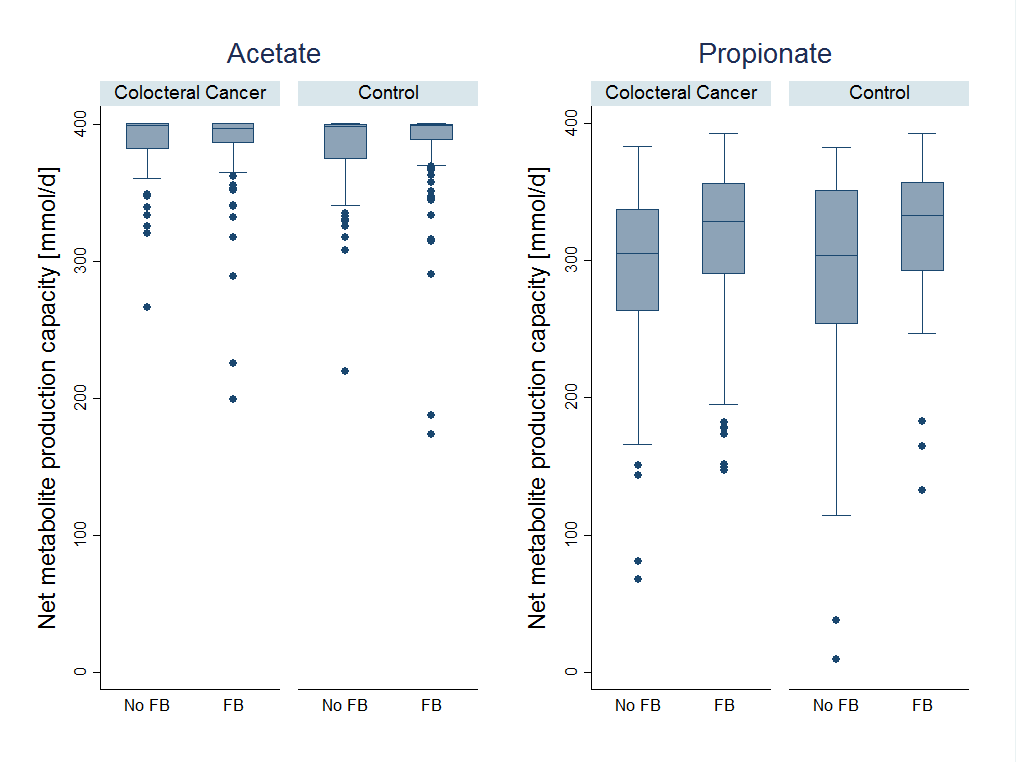


**Figure S1:** Box plots for net acetate and propionate production capacities for cases and controls across microbiomes with and without *Fusobacteria* presence. Communities with Fusobacteria had significantly higher net propionate production capacities (p=4.87e-07), while acetate production capacities were not significantly different (p=0.519). FB=Fusobacteria.
